# Associations of dietary biotin intake on anxiety and depression: findings from a population-based prospective cohort study

**DOI:** 10.3389/fnut.2025.1745340

**Published:** 2026-01-02

**Authors:** Yan Kong, Wei Xia, Tong Wang, Dongfeng Zhang

**Affiliations:** 1 Department of Epidemiology and Health Statistics, School of Public Health, Qingdao University, Qingdao, China; 2 Medical Record Management Center, the Affiliated Hospital of Qingdao University, Qingdao, Shandong, China

**Keywords:** anxiety, biotin, depression, epidemiology, inflammation

## Abstract

**Background:**

Some cross-sectional have found the negative association between dietary biotin intake and anxiety and depression symptoms. However, there is still a lack of cohort study in this field. So we conduct this prospective cohort study to investigate the association between dietary biotin intake and anxiety and depression, evaluate their dose–response relationship and the mediating role of inflammation in this process.

**Methods:**

A total of 144,439 UK Biobank participants without baseline anxiety or depression were included. Dietary biotin intake was derived from the 24-h Oxford WebQ data, and anxiety and depression were defined in accordance with the ICD-10 criteria. Cox proportional hazards models and restricted cubic splines (RCS) were used to evaluate longitudinal associations. The Karlson-Holm-Breen method was used to examine the mediating effect of inflammation.

**Results:**

A total of 144,439 participants were included in this study with a median follow-up of 14.06 years. Compared to Q1 group, dietary biotin intake was associated with a reduced risk of anxiety or depression (HR: 0.86 [0.82, 0.91] for Q2; HR: 0.84 [0.79, 0.88] for Q3; HR: 0.86 [0.81, 0.91] for Q4). Similar results were also found in anxiety, depression, and their comorbidity. RCS existed an approximately “L-shaped” dose–response relationship between biotin and both anxiety and depression. Except for depression and comorbidity, both single mediating indicators and composite mediating indicators played a partial mediating role in the course of this.

**Conclusion:**

Dietary biotin intake exhibited an approximately L-shaped nonlinear relationship with anxiety and depression risk, where risk decreased with increasing intake up to a moderate threshold, beyond which no further reduction was observed. This association may be partially mediated by inflammation.

## 1 Introduction

Anxiety and depression are the world’s most common mental disorders. Anxiety affected about 301 million people, and an estimated 5% of adults suffer from depression Globally (1, 2). Anxiety and depression remained significant contributors to the global disease burden, ranking 13th and 24th, respectively, among leading causes of disability-adjusted life-years (DALYs) (3). Anxiety and depression often co-occur and the treatment methods for the two are similar (4, 5). Nevertheless, approximately 75% of patients with anxiety and depression fail to receive effective treatment, particularly in low- and middle-income countries (6, 7). Therefore, it is necessary to investigate the modifiable risk factors and take effective corresponding prevention against them.

In recent years, some studies have found that micronutrients intake, such as vitamin D, vitamin B12, and magnesium exhibit protective effects against anxiety and depression (8–10). As a critical coenzyme for carboxylase enzymes, biotin (vitamin B7/H) involved in gluconeogenesis, lipid metabolism, and amino acid catabolism (11, 12). Biotin demonstrates regulatory functions in oxidative stress and inflammatory responses (13–15). Elevated inflammation demonstrated a robust correlation with an elevated risk of anxiety and depression (16–18). Biotin mitigates anxiety- and depression-like behaviors in rats through modulation of inflammatory pathways and oxidative stress (19). In population studies, a cross-sectional study suggested that Increased dietary biotin consumption was correlated with reduced symptoms of depression and anxiety (20). This study, however, was subject to certain limitations. First, the cross-sectional study was unable to verify longitudinal associations and the study outcomes were defined in the form of scales; second, this study did not explore the dose–response relationship between biotin and anxiety and depression; finally, did not investigate the mediating role of inflammation between them.

Therefore, we conducted this prospective longitudinal study from the UK Biobank to investigate: (1) the association between dietary biotin intake and anxiety and depression, (2) the dose–response relationship and (3) the potential mediating role of inflammation markers in this relationship.

## 2 Methods

### 2.1 Study population and design

UK Biobank is a population-based prospective cohort and biomedical database comprising half a million UK participants, with de-identified genetic, lifestyle and health information and biological samples (21). The baseline evaluation (2006–2010) enrolled 40–69 years old participants through a network of 22 standardized assessment centers, strategically located to ensure socioeconomic diversity, ethnic representation, and balanced urban–rural distribution within the cohort (22). Comprehensive phenotypic and genotypic data for UK Biobank participants are publicly accessible through the Data Showcase.^1^ The study protocol was approved by the North West Multicentre Research Ethics Committee, and all participants provided written informed consent. The UK Biobank application number of this research is 95,715.

**Figure 1:**
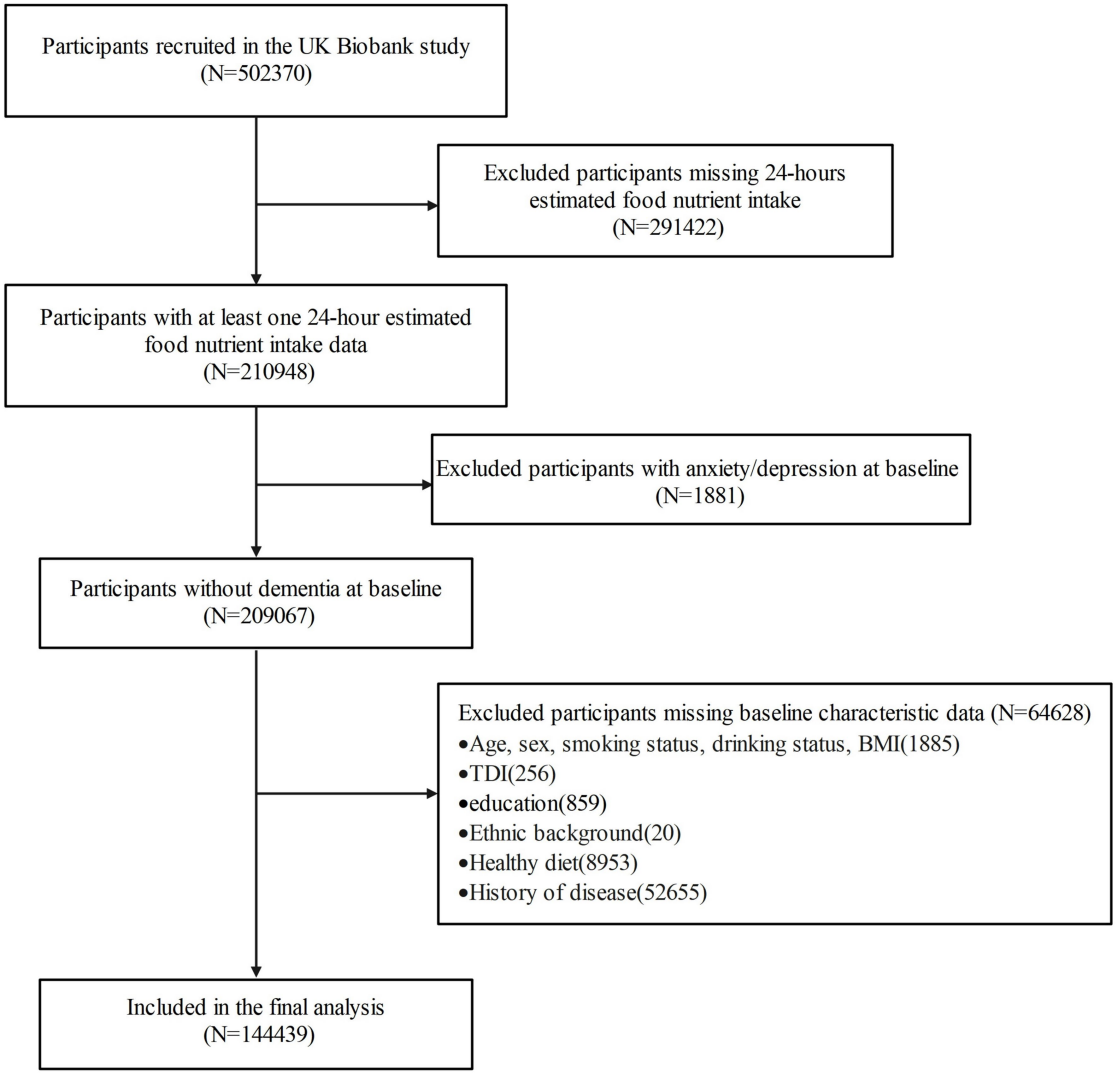
Selection of study participants in the UK Biobank.

Participants lacking the 24-h estimated intake of food nutrients (*n* = 291,422), those with anxiety or depression before baseline (*n* = 1881), and those without complete covariate information (*n* = 64,628) were excluded. Finally, 144,439 participants were eligible for inclusion criteria in this study (Figure 1). For mediation analysis of inflammatory markers, we further excluded 57,042 participants with missing immune biomarkers (*n* = 51,941) or extreme values (*n* = 5,101) (defined as <Q1–3 × IQR or >Q3 + 3 × IQR). Ultimately, a total of 87,397 participants with complete data on covariates, dietary biotin intake, inflammatory markers, and outcome variables were included in the mediation analysis.

### 2.2 Measurement of dietary biotin intake

Dietary biotin intake was quantified using the Oxford WebQ, an online 24-h recall diet questionnaire implemented during UK Biobank’s late recruitment phase (23). Participants (*n* = 210,948) reported consumption of 200 common food and beverage items, with automated nutrient calculation (including biotin) via linked composition databases (24). Dietary intake was quantified by multiplying self-reported consumption amounts by standardized portion sizes and corresponding nutrient values from the UK Nutrient Databank (2013) (25). For composite foods, nutrient intake was estimated by proportionally matching items to one or more food codes in the database. Vitamin and mineral supplements were not incorporated into the daily nutrient intake values generated. Repeated measures (4 email invitations at 3–4-month intervals) were averaged for individuals with multiple assessments.

### 2.3 Determination of anxiety and depression

The primary outcomes included (1) anxiety or depression, (2) anxiety, (3) depression, (4) comorbidity. According to the 10th Revision of the International Classification of Diseases (ICD-10), anxiety was defined by codes F40 and F41, while depression was defined by codes F32 and F33. The occurrence of anxiety, depression, or both was defined as anxiety or depression. The co-occurrence of anxiety and depression was defined as comorbidity. Death events were identified through the death registration information in the database. The follow-up time for each participant was measured from the date of attending assessment center until the earliest of: (1) anxiety and depression diagnosis, (2) death, (3) loss to follow-up, or (4) the date of the last data collection (30th September 2023), with a median follow-up of 14.06 years.

### 2.4 Definition of mediator

Inflammatory indices involved in various inflammatory processes were selected, including C-reactive protein (CRP), white blood cell counts, platelet counts, neutrophil counts, and glycoprotein acetyls (26). Extreme outlier values of these inflammatory indicators (defined as <Q1–3*IQR or >Q3 + 3*IQR) were excluded from the analysis. All individual immune markers were naturally log-transformed.

In addition to individual immune markers, composite inflammatory indices were calculated to comprehensively assess inflammatory status. The NLR, SII, and SIRI indices were calculated as neutrophil-to-lymphocyte ratio (NLR = Neutrophil/Lymphocyte), systemic immune-inflammation index (SII=Platele×Neutrophil/Lymphocyte), and systemic inflammation response index (SIRI=Neutrophil×Monocyte/Lymphocyte) (27–29). The low-grade chronic inflammation (INFLA) score integrates four inflammatory markers (CRP, WBC, platelets, NLR). Each component was scored from −4 (lowest decile) to +4 (highest decile), yielding a total range of −16 to +16, where higher values reflect greater inflammatory burden (30, 31).

### 2.5 Assessment of covariates

The collected sociodemographic factors included age, sex, ethnicity (White vs. others), education level (college/university degree vs. others), body mass index (BMI), and the Townsend deprivation index (calculated from participants’ residential postcode areas). Lifestyle factors comprised smoking status (never, former, current), drinking status (never, former, current), total energy intake, healthy diet (yes vs. no), and self-reported history of disease (hypertension, cardiovascular disease, diabetes, stroke). The Healthy Diet Score assessed dietary quality using six indicators: red meat, vegetables, fruit, fish, cereal intake, and urinary sodium excretion (32).

### 2.6 Statistical analysis

All participants were grouped by quartiles of dietary biotin intake. Continuous variables were presented as mean ± SD or median (IQR), while categorical variables were presented as number and percentage. Between-quartile differences were assessed using χ^2^ tests for categorical variables and ANOVA/Kruskal-Wallis tests for continuous variables, as appropriate.

The longitudinal relationship between dietary biotin intake and anxiety and depression was examined using Cox proportional hazards regression models. Age and sex were adjusted for in Model 1. Smoking status, drinking status, BMI, energy intake, ethnicity, TDI, education, and healthy diet were further adjusted in Model 2. Model 3 was additionally adjusted for the history of disease (hypertension, cardiovascular disease, diabetes, and stroke) based on Model 2. To assess potential nonlinear trends, the restricted cubic spline model (RCS) was employed with knots placed at the 25th (32.133 μg), 50th (40.540 μg), and 75th (50.765 μg) percentiles of biotin intake distribution. Hazard ratios with 95% confidence intervals were used to express all associations.

Considering the differences of age and sex in anxiety and depression, stratified analyses by age (≤60 vs. >60 years) and sex were conducted. Robustness was evaluated through comprehensive sensitivity analyses: (1) To address reverse causality, we excluded participants developing anxiety and depression within the first 2 years of follow-up. (2) To minimize bias from unreliable dietary reporting, we repeated all analyses after excluding participants with implausible energy intakes (<800 or >4,200 kcal/day for men, and <600 or >3,500 kcal/day for women). (3) Competing risk regression was employed, treating death and loss to follow-up as competing events. (4) Considering the potential variability in the participants’ diets, the analysis was specifically repeated for those individuals claiming that their diets were typical. (5) After the participants who took substances for anxiety and depression at the baseline were excluded, the associations between dietary biotin intake and anxiety and depression were explored once again.

To evaluate the role of inflammation in the association between biotin and anxiety and depression within the immune subset, we first assessed the relationship between biotin and the four outcomes using binary logistic regression. Subsequently, mediation analysis was performed with the Karlson-Holm-Breen (KHB) method in Stata, treating inflammatory markers as potential mediators (33). Firstly, we examined the individual mediating effects of each inflammatory marker on the biotin-anxiety and depression association. Subsequently, we constructed a combined mediation model including all inflammatory markers that showed significant mediation in initial simple models. In this study, the direct effect quantifies biotin’s unmediated influence on anxiety and depression, while indirect effects capture the proportion mediated through inflammatory pathways. The mediation proportion represents the ratio of indirect to total effects, with all analyses adjusted for full covariates (Model 3).

All statistical analyses were conducted using STATA/MP 18.0 and R 4.3.3, with two-tailed *p* < 0.05 was considered statistically significant.

## 3 Results

### 3.1 Characteristics of study participants

This study involved 144,493 participants, 54.7% of whom were female, with a median age of 58 years. During a median follow-up of 14.06 [13.42, 14.86] years, a total of 12,023 anxiety or depression cases, 6,881 anxiety cases, 7,870 depression cases and 2,728 comorbidity cases were recorded. Table 1 displayed the baseline characteristics grouped by dietary biotin intake quartiles. Participants in higher biotin quartiles were typically older, male, and more likely to have a healthy diet, higher energy intake, and advanced education. They also showed higher proportions of previous smokers, current drinkers, and histories of hypertension and cardiovascular disease, but lower BMI, fewer never-smokers, reduced alcohol abstainers, and lower stroke prevalence compared to lower biotin intake groups.

**Table 1:** Baseline characteristics of study population according to the quartiles of dietary biotin.

Characteristics	Overall	Dietary biotin intake^a^				*P*-value^b^
		First quartile (*n* = 36,110)	Second quartile (*n* = 36,111)	Third quartile (*n* = 36,108)	Forth quartile (*n* = 36,110)	
Age, years median (IQR)	58.0 [51.0, 63.0]	57.0 [50.0, 63.0]	59.0 [51.0, 63.0]	59.0 [52.0, 63.0]	59.0 [52.0, 63.0]	<0.001
Sex (%)						<0.001
Female	78,958(54.7)	23,431(64.9)	21,603(59.8)	18,989(52.6)	14,935(41.4)	
Male	65,481(45.3)	12,679(35.1)	14,508(40.2)	17,119(47.4)	21,175(58.6)	
Smoking status (%)						<0.001
Never	79,568(55.1)	20,684(57.3)	20,357(56.4)	19,957(55.3)	18,570(51.4)	
Previous	54,017 (37.4)	12,322(34.1)	13,303(36.8)	13,771(38.1)	14,621(40.5)	
Current	10,854 (7.5)	3,104(8.6)	2,451(6.8)	2,380(6.6)	2,919(8.1)	
Drinking status (%)						<0.001
Never	4,663(3.2)	1815(5.0)	1,068(3.0)	940(2.6)	840(2.3)	
Previous	4,797(3.3)	1,645(4.6)	1,140(3.2)	982(2.7)	1,030(2.9)	
Current	134,979(93.5)	32,650(90.4)	33,903(93.8)	34,186(94.7)	34,240(94.8)	
BMI, kg/m^2^ (mean ± SD)	27.3 ± 4.77	27.7 ± 5.08	27.2 ± 4.74	27.1 ± 4.57	27.3 ± 4.66	<0.001
Energy, kcal (mean ± SD)	2067 ± 604	1,623 ± 436	1930 ± 411	2,155 ± 448	2,558 ± 660	<0.001
TDI, median (IQR)	−2.33 [−3.73, 0.03]	−2.15 [−3.63, 0.39]	−2.40 [−3.76, −0.16]	−2.44[−3.80, −0.20]	−2.29 [−3.72, 0.11]	<0.001
Education level (%)						<0.001
College/university degree	60,055(41.6)	11,817(32.7)	14,744(40.8)	16,334(45.2)	17,160(47.5)	
Others	84,384(58.4)	24,293(67.3)	21,367(59.2)	19,774(54.8)	18,950(52.5)	
Ethnic (%)						<0.001
White	132,265(91.6)	32,901(91.1)	33,258(92.1)	33,265(92.1)	32,841(90.9)	
Others	12,174(8.4)	3,209(8.9)	2,853(7.9)	2,843(7.9)	3,269(9.1)	
Healthy diet (%)						<0.001
No	66,352(45.9)	19,323(53.5)	16,334(45.2)	15,065(41.7)	15,630(43.3)	
Yes	78,087(54.1)	16,787(46.5)	19,777(54.8)	21,043(58.3)	20,480(56.7)	
Hypertension (%)						0.095
No	97,739(67.7)	24,359(67.5)	24,572(68.0)	24,513(67.9)	24,295(67.3)	
Yes	46,700(32.3)	11,751(32.5)	11,539(32.0)	11,595(32.1)	11,815(32.7)	
Stroke (%)						0.004
No	142,560(98.7)	35,587(98.6)	35,620(98.6)	35,673(98.8)	35,680(98.8)	
Yes	1879(1.3)	523 (1.4)	491 (1.4)	435(1.2)	430(1.2)	
Diabetes (%)						<0.001
No	136,740(94.7)	34,079(94.4)	34,257(94.9)	34,312(95.0)	34,092(94.4)	
Yes	7,699(5.3)	2031(5.6)	1854(5.1)	1796(5.0)	2018(5.6)	
Cardiovascular disease (%)						0.002
No	110,120(76.2)	27,713(76.7)	27,638(76.5)	27,455(76.0)	27,314(75.6)	
Yes	34,319(23.8)	8,397(23.3)	8,473(23.5)	8,653(24.0)	8,796(24.4)	
C- reaction protein, mg/L (mean ± SD)	1.76 ± 1.62	1.92 ± 1.71	1.77 ± 1.62	1.69 ± 1.58	1.65 ± 1.56	<0.001
White blood cell count, *10^9/L (mean ± SD)	6.76 ± 1.59	6.87 ± 1.63	6.77 ± 1.58	6.72 ± 1.56	6.70 ± 1.58	<0.001
Platelet count, *10^9/L (mean ± SD)	245 ± 54.9	250 ± 56.4	246 ± 54.9	244 ± 54.1	240 ± 53.5	<0.001
Neutrophil count, *10^9/L (mean ± SD)	4.15 ± 1.26	4.23 ± 1.29	4.15 ± 1.26	4.11 ± 1.24	4.10 ± 1.25	<0.001
Glycoprotein Acetyls, mmol/L (mean ± SD)	0.79 ± 0.11	0.80 ± 0.12	0.79 ± 0.12	0.78 ± 0.11	0.78 ± 0.11	<0.001
Monocyte count, *10^9/L (mean ± SD)	0.47 ± 0.16	0.46 ± 0.15	0.47 ± 0.15	0.47 ± 0.15	0.48 ± 0.16	<0.001
Lymphocyte count, *10^9/L (mean ± SD)	1.94 ± 0.58	1.98 ± 0.60	1.94 ± 0.58	1.92 ± 0.57	1.91 ± 0.57	<0.001
NLR (mean ± SD)	2.30 ± 0.97	2.31 ± 1.00	2.30 ± 0.97	2.30 ± 0.96	2.31 ± 0.95	0.501
SII (mean ± SD)	564 ± 275	576 ± 283	566 ± 279	560 ± 271	555 ± 267	<0.001
SIRI (mean ± SD)	1.09 ± 0.62	1.07 ± 0.63	1.08 ± 0.61	1.09 ± 0.60	1.11 ± 0.62	<0.001
INFLA (mean ± SD)	0.03 ± 5.95	0.65 ± 6.00	0.10 ± 5.97	−0.21 ± 5.86	−0.43 ± 5.92	<0.001

TDI, Townsend deprivation index; BMI, Body mass index; SD, Standard deviation. ^a^Quartiles of dietary biotin intake. The cutoff points of the quartiles are 32.133, 40.540, 50.765. ^b^Analysis of variance or χ^2^ test where appropriate; NLR, Neutrophil-to-Lymphocyte Ratio; SII, Systemic Immune-inflammation Index; SIRI, Systemic Inflammation Response Index; INFLA, Low-grade chronic inflammation score.

### 3.2 Associations between dietary biotin intake and anxiety and depression risk

As presented in Table 2, dietary biotin intake demonstrated significant inverse associations with anxiety and depression across all three models.

**Table 2:** Associations between biotin and anxiety and depression (*n* = 144,439).

	Dietary biotin intake						
	First quartile	Second quartile		Third quartile		Forth quartile	
Anxiety or depression							
Number of cases/person- years	3,498/482,045	2,912/489,935		2,758/490,742		2,855/488,932	
		HR (95%CI)	*P*-value	HR (95%CI)	*P*-value	HR (95%CI)	*P*-value
Model 1^a^	1 (reference)	0.84(0.80, 0.88)	*<0.001*	0.82(0.78, 0.86)	*<0.001*	0.90(0.85, 0.94)	*<0.001*
Model 2^b^	1 (reference)	0.86(0.82, 0.91)	*<0.001*	0.84(0.79, 0.88)	*<0.001*	0.85(0.80, 0.91)	*<0.001*
Model 3^c^	1 (reference)	0.86(0.82, 0.91)	*<0.001*	0.84(0.79, 0.88)	*<0.001*	0.86(0.81, 0.91)	*<0.001*
Anxiety							
Number of cases/person- years	2003/492,698	1720/498,046		1,573/498,994		1,585/497,989	
		HR (95%CI)	*P*-value	HR (95%CI)	*P*-value	HR (95%CI)	*P*-value
Model 1^a^	1 (reference)	0.86(0.80, 0.92)	*<0.001*	0.81(0.76, 0.87)	*<0.001*	0.87(0.82, 0.93)	*<0.001*
Model 2^b^	1 (reference)	0.88(0.82, 0.94)	*<0.001*	0.82(0.76, 0.88)	*<0.001*	0.82(0.76, 0.89)	*<0.001*
Model 3^c^	1 (reference)	0.88(0.82, 0.94)	*<0.001*	0.82(0.77, 0.88)	*<0.001*	0.83(0.76, 0.90)	*<0.001*
Depression							
Number of cases/person- years	2,333/488,761	1,854/495,778		1,808/495,912		1,875/494,430	
		HR (95%CI)	*P*-value	HR (95%CI)	*P*-value	HR (95%CI)	*P*-value
Model 1	1 (reference)	0.81(0.76, 0.86)	*<0.001*	0.81(0.77, 0.87)	*<0.001*	0.89(0.83, 0.94)	*<0.001*
Model 2	1 (reference)	0.85(0.79, 0.90)	*<0.001*	0.85(0.80, 0.91)	*<0.001*	0.86(0.80, 0.93)	*<0.001*
Model 3	1 (reference)	0.85(0.80, 0.90)	*<0.001*	0.86(0.80, 0.92)	*<0.001*	0.87(0.81, 0.93)	*<0.001*
Comorbidity							
Number of cases/person- years	838/499,414	662/503,888		623/504,163		605/503,487	
		HR (95%CI)	*P*-value	HR (95%CI)	*P*-value	HR (95%CI)	*P*-value
Model 1	1 (reference)	0.80(0.73, 0.89)	*<0.001*	0.79(0.71, 0.88)	*<0.001*	0.81(0.73, 0.91)	*<0.001*
Model 2	1 (reference)	0.85(0.76, 0.94)	*0.002*	0.83(0.75, 0.93)	*0.002*	0.80(0.71, 0.91)	*0.001*
Model 3	1 (reference)	0.85(0.76, 0.94)	*0.003*	0.84(0.75, 0.94)	*0.002*	0.81(0.71, 0.91)	*0.001*

Results were presented HR and 95% CI. First quartile: 0.000 ≤ dietary biotin intake ≤32.133; second quartile: 32.133 < dietary biotin intake ≤ 40.540. Third quartile: 40.540 < dietary biotin intake ≤ 50.765; fourth quartile: 50.765 < dietary biotin intake ≤ 101.460. Italics represents *p* < 0.05. ^a^Model 1 was adjusted for age, sex. ^b^Model 2 was further adjusted for smoking status, drinking status, BMI, energy intake, ethnic background, TDI, education and healthy diet. ^c^Model 3 was adjusted for the same variables as in Model 2 and further for history of disease (hypertension, cardiovascular disease, diabetes, and stroke).

#### 3.2.1 Anxiety or depression

**Figure 2:**
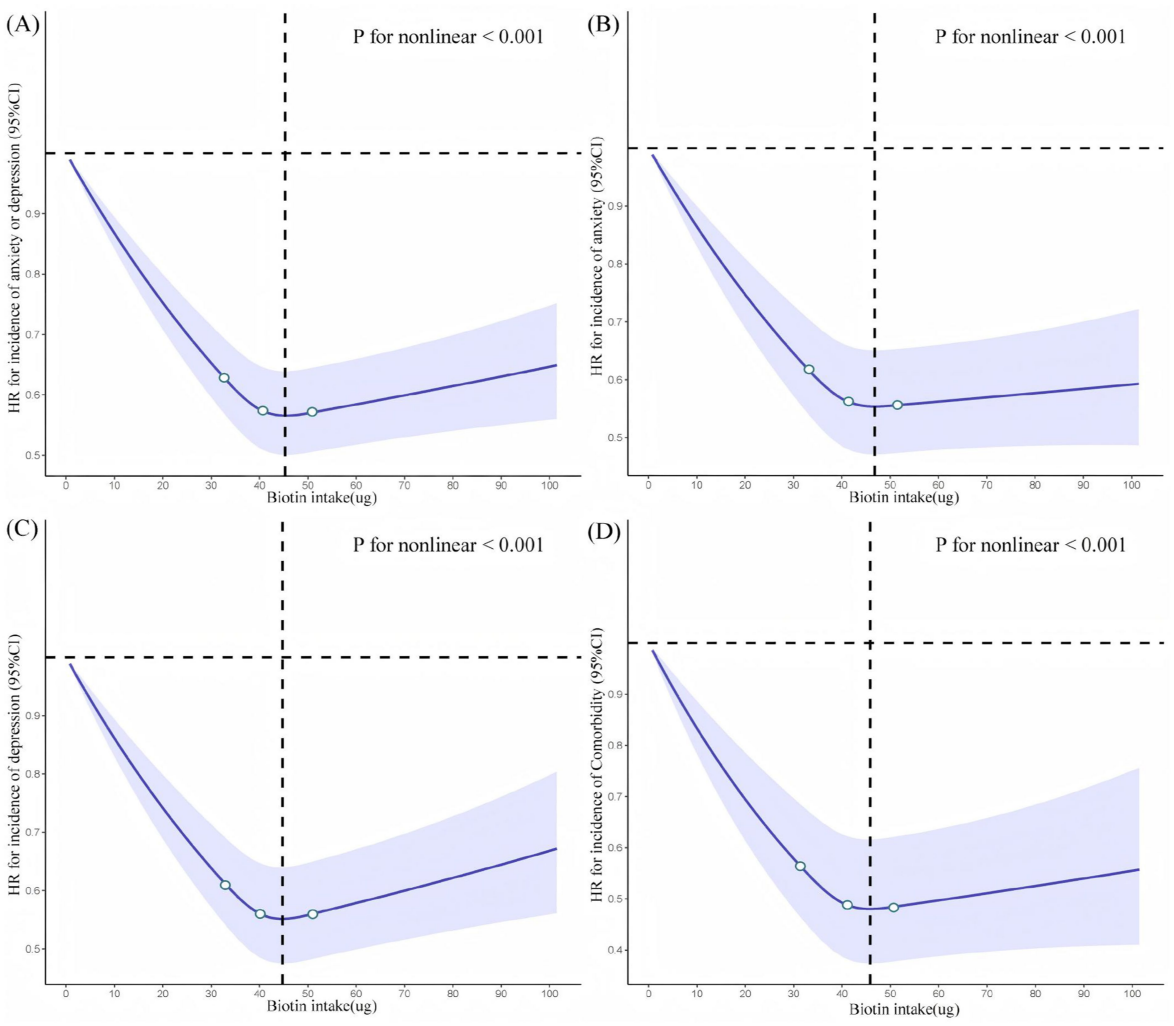
Restricted cubic spline for testing the hypothesis of nonlinear correlation between **(A)** Anxiety or depression. **(B)** Anxiety. **(C)** Depression. **(D)** Comorbidity and dietary biotin intake. Spline curves represent hazard ratios (HRs) adjusted for age, sex, smoking status, drinking status, BMI, energy intake, ethnic background, TDI, education and healthy diet, history of diseases (hypertension, cardiovascular disease, diabetes, and stroke). The solid lines are fitted based on Cox-proportional hazard models. The shaded areas show 95% confidential intervals (CIs). The black vertical line indicates the position where the curve inflection point occurs. The RCS were fitted with three knots placed at the 25th, 50th, and 75th percentiles of biotin intake (solid white points within a blue box). The smooth curve represents the overall modeled relationship, with all knots contributing to the shape of the spline.

During the follow-up period, a total of 12,023 individuals developed anxiety or depression. After adjusting for all covariates in Model 3, compared with the lowest intake group (Q1), biotin decreased the risk of anxiety or depression (HR: 0.86 [0.82, 0.91], *p* < 0.001 for Q2; HR: 0.84 [0.79, 0.88], *p* < 0.001 for Q3; HR: 0.86 [0.81, 0.91], *p* < 0.001 for Q4). RCS in Figure 2 and Supplementary Table S3 revealed an approximately “L-shaped nonlinear” trend (*P* for nonlinear < 0.001) between them, with the lowest risk at 45.174 μg/d.

#### 3.2.2 Anxiety

The incidence of anxiety was observed in a total of 6,881 individuals. In comparison with Q1, biotin was associated with a reduced risk of anxiety (HR: 0.88 [0.82, 0.94], *p* < 0.001 for Q2; HR: 0.82 [0.77, 0.88], *p* < 0.001 for Q3; HR: 0.83 [0.76, 0.90], *p* < 0.001 for Q4). RCS also exhibited an approximately “L-shaped” nonlinear relationship (*P* for nonlinear < 0.001), with the minimum risk observed at an intake of 47.394 μg/d.

#### 3.2.3 Depression

A total of 7,870 cases of depression onset were identified. Relative to Q1, biotin intake was linked to a lower risk of depression (HR: 0.85 [0.80, 0.90], *p* < 0.001 for Q2; HR: 0.86 [0.80, 0.92], *p* < 0.001 for Q3; HR: 0.87 [0.81, 0.93], *p* < 0.001 for Q4). RCS revealed an approximately “L-shaped” nonlinear trend (*P* for nonlinear < 0.001) in this association, with the lowest risk observed at an intake level of 45.174 μg/d.

#### 3.2.4 Comorbidity

Throughout the follow-up phase, 2,728 new cases of comorbidity, referring to the co-occurrence of anxiety and depression, were recorded. For comorbidity, compared with Q1, biotin reduced the risk of comorbidity (HR: 0.85 [0.76, 0.94], *p* = 0.003 for Q2; HR: 0.84 [0.75, 0.94], *p* = 0.002 for Q3; HR: 0.81 [0.71, 0.91], *p* = 0.001 for Q4), and the RCS also showed an approximately “L-shaped” nonlinear relationship, with the lowest risk at 46.284 μg/d.

### 3.3 Mediating role of inflammatory factors

**Figure 3:**
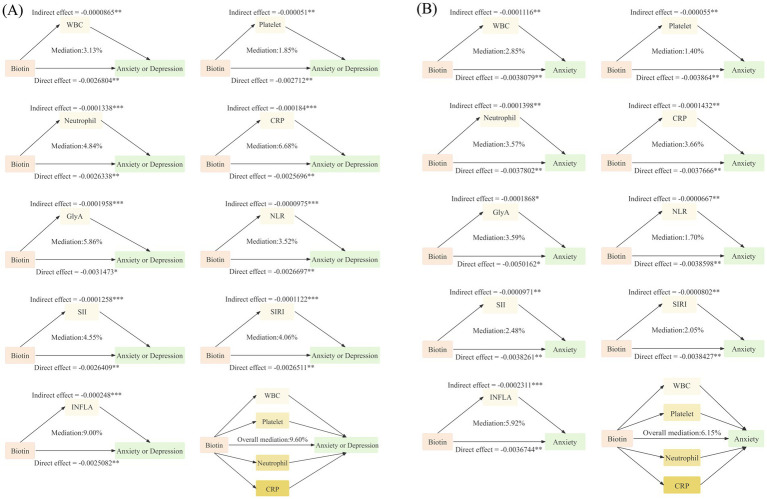
Mediation analyses with inflammatory indicators between the association of dietary biotin intake and **(A)** anxiety or depression, **(B)** anxiety among immune subset (*N* = 87,397). Adjusted for age, sex, smoking status, drinking status, BMI, energy intake, ethnic background, TDI, education and healthy diet, history of disease (hypertension, cardiovascular disease, diabetes, and stroke). *p < 0.05; **p < 0.01; ***p < 0.001. WBC, white blood cell; CRP, C-reaction protein; NLR, Neutrophil-to-Lymphocyte Ratio; SII, Systemic Immune-inflammation Index; SIRI, Systemic Inflammation Response Index; INFLA, Low-grade chronic inflammation score.

Figure 3 illustrates the significant mediating effects and their proportions of inflammatory markers in the biotin-anxiety and depression. For anxiety or depression, individual indices WBC, platelets, neutrophils, CRP, GlyA and composite indices NLR, SII, SIRI, INFLA all showed significant mediating effects, with intermediate ratios of 3.13, 1.85, 4.84, 6.68, 5.86, and 3.52%, 4.55, 4.06, 9.00%, respectively. All significant individual inflammatory markers were integrated into the mediating model, and the overall mediating effect was statistically significant, with an intermediate ratio of 9.60%.

Similarly, for anxiety specifically, mediating effects were observed through WBC, platelets, neutrophils, CRP, and GlyA, with composite indices NLR, SII, SIRI, and INFLA, with intermediate ratios of 2.85, 1.40, 3.57, 3.66, 3.59, and 1.70%, 2.48, 2.05, 5.92%, respectively. The overall mediation proportion of all significant individual inflammatory markers was significant, with an intermediate ratio of 6.15%. However, no mediating effects of inflammatory markers were observed for depression or comorbidity.

More detailed results were presented in Supplementary Tables S1, S2.

### 3.4 Subgroup analyses

Subgroup analyses (Supplementary Figure S1) were conducted stratifying by age (≤60 or >60 years) and sex (female or male). In each of these subgroups, participants were further divided into quartiles according to biotin intake levels. In the four outcomes of anxiety or depression, anxiety, depression, and comorbidity, the results of subgroup analyses stratified by age all showed the protective effect of biotin on them, except for the Q2 in the comorbidity. However, when stratified by sex, the protective effect of biotin in males was only observed in the higher quartiles (Q3 and Q4) across all four outcomes.

### 3.5 Sensitivity analyses

Sensitivity analyses (Supplementary Tables S4–S8 and Supplementary Figures S2–S5) confirmed the robustness of these findings. Reanalyses confirmed that the results remained stable after excluding participants who developed anxiety or depression with less than 2 years of follow-up (Supplementary Table S4 and Supplementary Figure S2). Participants with extreme energy intake were excluded, and the resulting findings were consistent with those from the full population analysis (Supplementary Table S5 and Supplementary Figure S3). In competing risk models of death (Supplementary Table S6), the association between biotin and the risk of the four outcomes showed no significant changes. Analyses of participants with typical diets yielded results that aligned with the primary findings (Supplementary Table S7 and Supplementary Figure S4). Additionally, reanalyses excluding participants who had taken substances for anxiety or depression at baseline also demonstrated stable results (Supplementary Table S8 and Supplementary Figure S5).

## 4 Discussion

Leveraging the UK Biobank’s large-scale longitudinal data, we identified dietary biotin intake could reduce the risk of anxiety or depression, anxiety, depression and comorbidity with a median follow-up of 14.06 years. In the adjusted RCS model, the relationship between biotin and risk of four outcomes were all nonlinear and the trends were approximately L-shaped, with the inflection points at 45.174, 47.394, 45.174, and 46.284 μg/d, respectively. Subgroup analyses demonstrated consistent outcomes across different age strata and in females, except that a higher biotin intake was required for the protective effect on males. The stability of the study results was supported by sensitivity analyses. This suggested that higher dietary biotin intake may act as a protective factor against anxiety and depression. Mediation analyses confirmed that inflammation played a mediating role, as evidenced by both single biomarkers and composite indices.

Currently, only one cross-sectional study with a sample size of 7,387 has explored the relationship between dietary biotin and anxiety, depression in human populations (20). This study suggested that higher intake of biotin was associated with lower odds of anxiety and depression symptoms, which were consistent with our study. Another descriptive study on Spanish children aged 6–9 showed a negative association between children with depressive symptoms and non-depressive symptoms for biotin (34).

When the dose–response relationship was further analyzed, the relationships between biotin and anxiety and depression were found not to be a simple linear one, but an approximately L-shaped one. In our study, the inflection point of biotin was between 45 and 48 μg/day, which was slightly higher than the adequate intake (AI) for adults recommended by the European Food Safety Authority (EFSA), which is 40 μg/day (35). It was worth noting that our sex-stratified analysis showed that the biotin inflection point occurred at 40–42 μg/day in males and 45–48 μg/day in females, indicating that higher biotin levels are required to exert a protective effect on the four outcomes in males compared with females. RCS indicated the difference in inflection points between males and females (Supplementary Figure S6). In fact, males showed higher levels of oxidative stress and baseline inflammation than females (36). Therefore, males may need higher intake of biotin to adequately activate anti-inflammatory and antioxidant pathways against anxiety and depression. The above findings reveal a difference in the inflection points of biotin intake between males and females: males have a higher inflection point than females, and both are higher than the AI. Based on these findings, the present study may offer preliminary insights for informing future recommendations regarding Recommended Nutrient Intake (RNI). It also suggests that consideration could be given to setting a higher RNI for males compared to females.

The biological mechanisms by which dietary biotin reduces the risk of anxiety and depression may involve the following aspects: Biotin serves as an essential coenzyme of five carboxylases for five carboxylases: acetyl CoA carboxylase 1 (ACC1) and 2 (ACC2), methylcrotonyl CoA carboxylase (MCC), propionyl CoA carboxylase (PCC), and pyruvate carboxylase (PC), playing pivotal roles in regulations of ATP production, Oxidative stress, immunological and inflammatory functions (11, 12, 14, 15, 37, 38). Biotin showed an inverse correlation with inflammation, which aligns with previous research. In a state of biotin deficiency, phenomena such as enhanced activation of immune cell signaling pathways, increased levels of pro-inflammatory cytokines, and decreased levels of anti-inflammatory factors occur (13, 39, 40). Systemic inflammation triggers neuroinflammation, which can exert direct and indirect neurotoxic effects, thereby potentially contributing to the development of related psychiatric symptoms (41, 42). Our study provides further understanding for this through mediation analyses of both single and composite inflammatory markers. Additionally, biotin can also affect the metabolism of branched-chain amino acids (BCAAs) by influencing PCC, thereby increasing the risk of depression (43, 44). Biotin may influence mitochondrial function and energy metabolism through its regulatory role in PC and ACC to affect the onset of anxiety and depression (45). The multiple biological mechanisms by which biotin reduce the risk of anxiety and depression may collectively explain why the mediating role of inflammation remains meaningful, although limited in its proportional contribution.

Indeed, lifestyle factors such as physical activity, smoking, drinking, and sleep patterns can simultaneously influence both systemic inflammation levels and mental health status (46–50). Additionally, specific clinical conditions (such as autoimmune diseases) and medication use (such as anti-inflammatory drugs, psychotropic medications) may interfere with the biotin-inflammation-mental health association through immunomodulatory or neuroendocrine pathways (51–53). Furthermore, broader psychosocial environments (such as stress, social support) and dietary patterns may also serve as common variables affecting the non-linear relationships in this study (54–57). Future research could further explain these complex interactions by incorporating more comprehensive lifestyle assessments, medication use history, multi-nutrient joint analyses, and prospective dynamic monitoring.

This study has several advantages. First of all, to our knowledge, this is the first longitudinal study based on a large-scale prospective cohort design that explores the relationship between dietary biotin intake and anxiety and depression and their comorbidity, with the research results having higher statistical power and stronger extrapolability. Secondly, by exploring the dose–response relationship between biotin and anxiety and depression, this study can quantify the degree of their association, clarify the strength and direction of the causal effect. Moreover, it can provide a reference for determining the standard of recommended intake and provide a basis for revealing potential biological mechanisms. Thirdly, this study explored the mediating role of inflammation, providing more abundant evidence for the explanation of the mechanism. Fourthly, our outcomes were diagnosed based on the ICD-10 criteria, which ensures the objectivity and standardization of outcomes, enhances the comparability and the persuasiveness of the research conclusions. Finally, in the analysis process, this study fully adjusted for various confounding factors, and conducted sufficient subgroup analyses and sensitivity analyses, which enhanced the robustness of the research results.

However, this study still has certain limitations. Although the 24-h dietary recall diet questionnaire was used to define biotin intake levels, which can directly reflect the actual recent biotin intake status, this method may still introduce recall bias due to the memory bias of the participants. To address this, this study improved the stability of biotin intake level assessment by averaging the results of multiple questionnaires, thereby minimizing the impact of recall bias on the outcomes. Then, the participants in the UK Biobank are mainly White people, so caution should be exercised when extrapolating the research results to other populations. In addition, the study only explored the mediating role of inflammatory indicators, and the analysis results suggest that there are other mechanisms underlying the effect of biotin on anxiety and depression that still need to be investigated. Moreover, study outcomes defined solely using ICD-10 codes may not capture all affected individuals, particularly those from populations with limited healthcare access, lower socioeconomic status, or unfavorable lifestyle patterns. As a result, the protective effect of biotin could be underestimated, and both internal validity and generalizability of the findings may be compromised.

## 5 Conclusion

In conclusion, our findings indicated that dietary biotin intake exhibited an approximately ‘L-shaped’ nonlinear relationship with the risk of anxiety and depression. The risk decreased with increasing intake up to a threshold approximately at moderate intake levels, beyond which further increases in intake do not confer additional risk reduction. Inflammation might play a partial mediating role in this association. An appropriate amount of dietary biotin intake could have contributed to reducing the risk of developing anxiety and depression.

## Data Availability

The datasets presented in this study can be found in online repositories. The names of the repository/repositories and accession number(s) can be found in the article/Supplementary material.

## Footnotes

1
http://www.ukbiobank.ac.uk/
