# Supplementary material for: Associations of dietary biotin intake on anxiety and depression: findings from a population-based prospective cohort study
Source: Front Nutr. 2026 Jan 2;12:1745340. doi: 10.3389/fnut.2025.1745340 (PMC12807950; doi:10.3389/fnut.2025.1745340)
Supplement: Supplementary file 1 [file Table_1.DOCX]

Supplementary Material

**Table S1** Mediation analysis of inflammatory factors in the association of dietary biotin intake and anxiety or depression.

| **Mediator** | **Indirect effect**  **(coefficient,95%CI)** | **Direct effect**  **(coefficient,95%CI)** | **Total effect**  **(coefficient,95%CI)** | **Mediation proportion(%)** |
| --- | --- | --- | --- | --- |
| WBC | -0.0000865  (-0.0001475,-0.0000255)** | -0.0026804  (-0.0045123,-0.0008485)** | -0.0027669  (-0.0045978,-0.000936)** | 3.13 |
| platelets | -0.000051  (-0.0000861,-0.0000159)** | -0.002712  (-0.0045435,-0.0008806)** | -0.002763  (-0.0045943,-0.0009318)** | 1.85 |
| neutrophils | -0.0001338  (-0.0001995,-0.0000682)*** | -0.0026338  (-0.0044654,-0.0008022)** | -0.0027677  (-0.0045983,-0.0009371)** | 4.84 |
| CRP | -0.000184  (-0.0002642,-0.0001039)*** | -0.0025696  (-0.0043997,-0.0007394)** | -0.0027536  (-0.0045819,-0.0009253)** | 6.68 |
| GlyA | -0.0001958  (-0.0003132,-0.0000783)** | -0.0031473  (-0.0061788,-0.0001158)* | -0.0033431  (-0.0063722,-0.000314)* | 5.86 |
| NLR | -0.0000975  (-0.0001444,-0.0000505)*** | -0.0026697  (-0.0045009,-0.0008386)** | -0.0027672  (-0.0045979,-0.0009365)** | 3.52 |
| SII | -0.0001258  (-0.0001787,-0.000073)*** | -0.0026409  (-0.0044721,-0.0008096)** | -0.0027667  (-0.0045974,-0.0009359)** | 4.55 |
| SIRI | -0.0001122  (-0.0001627,-0.0000618)*** | -0.0026511  (-0.0044827, -0.0008194)** | -0.0027633  (-0.0045944,-0.0009322)** | 4.06 |
| INFLA | -0.000248  (-0.0003343,-0.0001618)*** | -0.0025082  (-0.0043392, -0.0006772) ** | -0.0027562  (-0.0045854,-0.0009271)** | 9.00 |

Mediation analyses with inflammatory indicators between the association of dietary biotin intake and anxiety or depression among immune subset (N=87397). Adjusted for age, sex, smoking status, drinking status, BMI, energy intake, ethnic background, TDI, education and healthy diet, history of disease(hypertension, cardiovascular disease, diabetes, and stroke).

**P* < 0.05;***P* < 0.01;****P* <0.001

Abbreviations:WBC: white blood cell; CRP:C-reaction protein;

NLR:Neutrophil-to-Lymphocyte Ratio;

SII:Systemic Immune-inflammation Index;

SIRI:Systemic Inflammation Response Index;

INFLA:Low-grade chronic inflammation score.

**Table S2** Mediation analysis of inflammatory factors in the association of dietary biotin intake and anxiety.

| **Mediator** | **Indirect effect**  **(coefficient,95%CI)** | **Direct effect**  **(coefficient,95%CI)** | **Total effect**  **(coefficient,95%CI)** | **Mediation proportion(%)** |
| --- | --- | --- | --- | --- |
| WBC | -0.0001116  (-0.0001902,-0.000033)** | -0.0038079  (-0.00622,-0.0013958)** | -0.0039195  (-0.0063302,-0.0015088)** | 2.85 |
| platelets | -0.000055  (-0.0000963,-0.0000138)** | -0.003864  (-0.0062756,-0.0014525)** | -0.0039191  (-0.0063304,-0.0015078)** | 1.40 |
| neutrophils | -0.0001398  (-0.000222,-0.0000577)** | -0.0037802  (-0.0061919,-0.0013686)** | -0.0039201  (-0.0063304,-0.0015098)** | 3.57 |
| CRP | -0.0001432  (-0.0002417,-0.0000448)** | -0.0037666  (-0.0061774,-0.0013559)** | -0.0039099  (-0.0063182,-0.0015016)** | 3.66 |
| GlyA | -0.0001868  (-0.0003302,-0.0000434)* | -0.0050162  (-0.0090541,-0.0009783)* | -0.005203  (-0.0092374,-0.0011686)* | 3.59 |
| NLR | -0. 0000667  (-0.0001177,-0.0000158)* | -0.0038598  (-0.0062712,-0.0014484)** | -0.0039265  (-0.0063374,-0.0015156) | 1.70 |
| SII | -0.0000971  (-0.0001549,-0.0000393)** | -0.0038261  (-0.0062374,-0.0014147)** | -0.0039232  (-0.0063339,-0.0015125)** | 2.48 |
| SIRI | -0.0000802  (-0.0001366,-0.0000239)** | -0.0038427  (-0.0062546,-0.0014308)** | -0.0039229  (-0.0063341,-0.0015117)** | 2.05 |
| INFLA | -0.0002311  (-0.0003368,-0.0001255)*** | -0.0036744  (-0.0060854,-0.0012634)** | -0.0039055  (-0.006314,-0.001497)** | 5.92 |

Mediation analyses with inflammatory indicators between the association of dietary biotin intake and anxiety among immune subset (N=87397). Adjusted for age, sex, smoking status, drinking status, BMI, energy intake, ethnic background, TDI, education and healthy diet, history of disease(hypertension, cardiovascular disease, diabetes, and stroke).

**P* < 0.05;***P* < 0.01;****P* <0.001

Abbreviations:WBC: white blood cell; CRP:C-reaction protein;

NLR:Neutrophil-to-Lymphocyte Ratio;

SII:Systemic Immune-inflammation Index;

SIRI:Systemic Inflammation Response Index;

INFLA:Low-grade chronic inflammation score.

**Table S3** HR and its 95% Confidence Interval for the Restricted Cubic Spline (n=144,439).

| **Biotin^a^** | **Anxiety or Depression^b^** | | | **Anxiety^b^** | | | **Depression^b^** | | | **Comorbidity^b^** | | |
| --- | --- | --- | --- | --- | --- | --- | --- | --- | --- | --- | --- | --- |
|  | **yhat** | **lower** | **upper** | **yhat** | **lower** | **upper** | **yhat** | **lower** | **upper** | **yhat** | **lower** | **upper** |
| 0.769 | 0.992 | 0.990 | 0.994 | 0.992 | 0.989 | 0.995 | 0.991 | 0.989 | 0.994 | 0.989 | 0.984 | 0.993 |
| 5.829 | 0.942 | 0.927 | 0.958 | 0.941 | 0.921 | 0.962 | 0.935 | 0.917 | 0.954 | 0.918 | 0.888 | 0.949 |
| 10.889 | 0.894 | 0.867 | 0.922 | 0.893 | 0.857 | 0.930 | 0.883 | 0.851 | 0.916 | 0.852 | 0.801 | 0.907 |
| 15.949 | 0.849 | 0.812 | 0.888 | 0.847 | 0.798 | 0.899 | 0.833 | 0.789 | 0.880 | 0.791 | 0.722 | 0.867 |
| 21.009 | 0.806 | 0.760 | 0.855 | 0.804 | 0.743 | 0.869 | 0.786 | 0.732 | 0.845 | 0.735 | 0.651 | 0.828 |
| 26.068 | 0.765 | 0.711 | 0.824 | 0.763 | 0.692 | 0.840 | 0.742 | 0.679 | 0.811 | 0.682 | 0.588 | 0.792 |
| 31.128 | 0.727 | 0.666 | 0.793 | 0.724 | 0.644 | 0.813 | 0.700 | 0.629 | 0.779 | 0.633 | 0.530 | 0.756 |
| 32.133 | 0.719 | 0.657 | 0.787 | 0.716 | 0.635 | 0.807 | 0.692 | 0.620 | 0.773 | 0.624 | 0.519 | 0.750 |
| 36.188 | 0.692 | 0.625 | 0.765 | 0.688 | 0.601 | 0.787 | 0.663 | 0.586 | 0.750 | 0.589 | 0.480 | 0.724 |
| 40.540 | 0.673 | 0.603 | 0.751 | 0.668 | 0.578 | 0.772 | 0.643 | 0.562 | 0.735 | 0.565 | 0.452 | 0.706 |
| 41.248 | 0.672 | 0.602 | 0.750 | 0.667 | 0.576 | 0.772 | 0.642 | 0.561 | 0.734 | 0.564 | 0.451 | 0.705 |
| 46.308 | 0.676 | 0.605 | 0.756 | 0.668 | 0.576 | 0.774 | 0.647 | 0.565 | 0.741 | 0.564 | 0.450 | 0.707 |
| 50.765 | 0.691 | 0.620 | 0.771 | 0.680 | 0.588 | 0.786 | 0.664 | 0.581 | 0.758 | 0.577 | 0.462 | 0.720 |
| 51.368 | 0.693 | 0.622 | 0.773 | 0.681 | 0.590 | 0.787 | 0.666 | 0.583 | 0.760 | 0.578 | 0.464 | 0.721 |
| 56.427 | 0.713 | 0.641 | 0.793 | 0.696 | 0.605 | 0.802 | 0.687 | 0.604 | 0.783 | 0.595 | 0.480 | 0.738 |
| 61.487 | 0.732 | 0.660 | 0.813 | 0.712 | 0.620 | 0.817 | 0.709 | 0.624 | 0.806 | 0.612 | 0.495 | 0.757 |
| 66.547 | 0.753 | 0.679 | 0.835 | 0.727 | 0.634 | 0.834 | 0.732 | 0.645 | 0.831 | 0.630 | 0.510 | 0.777 |
| 71.101 | 0.772 | 0.696 | 0.856 | 0.742 | 0.647 | 0.850 | 0.754 | 0.664 | 0.855 | 0.646 | 0.523 | 0.796 |
| 76.161 | 0.793 | 0.715 | 0.880 | 0.758 | 0.661 | 0.870 | 0.778 | 0.686 | 0.882 | 0.664 | 0.538 | 0.820 |
| 81.220 | 0.815 | 0.734 | 0.905 | 0.775 | 0.674 | 0.890 | 0.803 | 0.707 | 0.912 | 0.683 | 0.552 | 0.846 |
| 86.280 | 0.838 | 0.753 | 0.932 | 0.792 | 0.687 | 0.912 | 0.829 | 0.728 | 0.943 | 0.703 | 0.565 | 0.874 |
| 91.340 | 0.861 | 0.773 | 0.960 | 0.809 | 0.700 | 0.935 | 0.856 | 0.749 | 0.977 | 0.723 | 0.578 | 0.904 |
| 96.400 | 0.885 | 0.792 | 0.990 | 0.827 | 0.712 | 0.960 | 0.883 | 0.771 | 1.012 | 0.743 | 0.590 | 0.936 |
| 101.460 | 0.910 | 0.811 | 1.022 | 0.845 | 0.724 | 0.986 | 0.912 | 0.792 | 1.049 | 0.765 | 0.602 | 0.971 |

HR and its 95% Confidence Interval for the Restricted Cubic Spline of dietary biotin intake and anxiety among full population (N=144,439). The RCS were fitted with three knots placed at the 25^th^ (32.133μg), 50^th^ (40.540μg), and 75^th^ (50.765μg) percentiles of biotin intake. Adjusted for age, sex, smoking status, drinking status, BMI, energy intake, ethnic background, TDI, education and healthy diet, history of disease (hypertension, cardiovascular disease, diabetes, and stroke).

^a^ Biotin intake(ug)

^b^ HR and the upper and lower limits of the 95% confidence interval

**Table S4.** Associations between dietary biotin intake and anxiety and depression after excluding participants who developed anxiety and depression within the first 2 years(n = 143,567).^a^

|  | **Dietary biotin intake** | | | | | | | | |
| --- | --- | --- | --- | --- | --- | --- | --- | --- | --- |
|  | **First quartile** | **Second quartile**  **HR (95%CI), *P*-value** | | | **Third quartile**  **HR (95%CI),*P*-value** | | | **Forth quartile**  **HR (95%CI),*P*-value** | |
| Anxiety or Depression disorders |  |  | | |  | | |  | |
| Number of cases/person- years | 3219 / 482624 | 2717 / 489399 | | | 2578 / 489987 | | | 2637 / 488701 | |
| Multivariate model^b^ | 1 (reference) | 0.87(0.83,0.92) | **<0.001** | 0.85(0.81,0.90) | | **<0.001** | 0.86(0.81,0.92) | | **<0.001** |
| Anxiety |  |  | | |  | | |  | |
| Number of cases/person- years | 1861 / 491223 | 1628 / 496114 | | | 1501 / 496768 | | | 1496 / 496017 | |
| Multivariate model^b^ | 1 (reference) | 0.90(0.84,0.96) | **0.002** | | 0.85(0.79,0.91) | **<0.001** | | 0.85(0.78,0.92) | **<0.001** |
| Depression |  |  | | |  | | |  | |
| Number of cases/person- years | 2106 / 488648 | 1707 / 494562 | | | 1658 / 494725 | | | 1693 / 493701 | |
| Multivariate model^b^ | 1 (reference) | 0.86(0.81,0.92) | **<0.001** | | 0.87(0.81,0.93) | **<0.001** | | 0.87(0.81,0.94) | **<0.001** |
| Comorbidity |  |  | | |  | | |  | |
| Number of cases/person- years | 748 / 497247 | 618 / 501277 | | | 581 / 501507 | | | 552 / 501017 | |
| Multivariate model^b^ | 1 (reference) | 0.89(0.80,0.99) | **0.044** | | 0.88(0.79,0.99) | **0.038** | | 0.84(0.73,0.96) | **0.011** |

a Results were presented HR and 95% CI

b Model was was adjusted age, sex, smoking status, drinking status, BMI, energy intake, ethnic background, TDI, education and healthy diet, history of

disease (hypertension, cardiovascular disease, diabetes, and stroke).

First quartile: 0.000 ≤ dietary biotin intake ≤ 32.150; second quartile: 32.150 < dietary biotin intake ≤ 40.549; third quartile: 40.549 < dietary biotin intake ≤

50.765; fourth quartile: 50.765 < dietary biotin intake ≤ 101.440.

Bold represents P-value <0.05.

**Table S5.** Associations Between dietary biotin intake and anxiety and depression excluding unreliable data on energy intake(n = 142,674).^a^

|  | **Dietary biotin intake** | | | | | | | |
| --- | --- | --- | --- | --- | --- | --- | --- | --- |
|  | **First quartile** | **Second quartile**  **HR (95%CI), *P*-value** | | **Third quartile**  **HR (95%CI),*P*-value** | | | **Forth quartile**  **HR (95%CI),*P*-value** | |
| Anxiety or Depression disorders |  |  | |  | | |  | |
| Number of cases/person- years | 3448 / 476491 | 2869 / 483970 | | 2722 / 484808 | | | 2764 / 483656 | |
| Multivariate model^b^ | 1 (reference) | 0.86(0.82,0.90) | **<0.001** | 0.84(0.79,0.88) | **<0.001** | 0.85(0.80,0.90) | | **<0.001** |
| Anxiety |  |  | |  | | |  | |
| Number of cases/person- years | 1974 / 4486978 | 1696 / 4919348 | | 1554 / 492952 | | | 1541 / 492301 | |
| Multivariate model^b^ | 1 (reference) | 0.88(0.82,0.94) | **<0.001** | 0.82(0.76,0.88) | **<0.001** | | 0.82(0.76,0.89) | **<0.001** |
| Depression |  |  | |  | | |  | |
| Number of cases/person- years | 2295 / 483099 | 1826 / 489735 | | 1780 / 489940 | | | 1804 / 488978 | |
| Multivariate model^b^ | 1 (reference) | 0.84(0.79,0.90) | **<0.001** | 0.85(0.80,0.91) | **<0.001** | | 0.86(0.80,0.93) | **<0.001** |
| Comorbidity |  |  | |  | | |  | |
| Number of cases/person- years | 821 / 493586 | 653 / 497700 | | 612 / 498084 | | | 581 / 497623 | |
| Multivariate model^b^ | 1 (reference) | 0.86(0.77,0.96) | **0.005** | 0.85(0.75,0.95) | **0.004** | | 0.81(0.71,0.92) | **0.002** |

a Results were presented HR and 95% CI

b Model was was adjustedage, sex, smoking status, drinking status, BMI, energy intake, ethnic background, TDI, education and healthy diet, history of

disease (hypertension, cardiovascular disease, diabetes, and stroke).

First quartile: 1.872 ≤ dietary biotin intake ≤ 32.135; second quartile: 32.135 < dietary biotin intake ≤ 40.442; third quartile: 40.442 < dietary biotin intake ≤

50.492; fourth quartile: 50.492< dietary biotin intake ≤ 97.421.

Bold represents P-value <0.05.

**Table S6.** Association between dietary biotin intake and anxiety and depression: results from competing risk regression models.^a^

|  | **Dietary biotin intake** | | | |
| --- | --- | --- | --- | --- |
|  | **First quartile** | **Second quartile**  **HR (95%CI)** | **Third quartile**  **HR (95%CI)** | **Forth quartile**  **HR (95%CI)** |
| Anxiety or Depression disorders |  |  |  |  |
| Multivariate model^b^ | 1 (reference) | 0.86(0.82,0.91) | 0.84(0.80.0.88) | 0.86(0.81,0.91) |
| Anxiety |  |  |  |  |
| Multivariate model^b^ | 1 (reference) | 0.88(0.82,0.94) | 0.82(0.77,0.88) | 0.83(0.76,0.90) |
| Depression |  |  |  |  |
| Multivariate model^b^ | 1 (reference) | 0.84(0.79,0.90) | 0.85(0.80,0.91) | 0.87(0.81,0.93) |
| Comorbidity |  |  |  |  |
| Multivariate model^b^ | 1 (reference) | 0.86(0.82,0.91) | 0.84(0.79,0.88) | 0.86(0.81,0.91) |

a Results were presented HR and 95% CI

b Model was was adjustedage, sex, smoking status, drinking status, BMI, energy intake, ethnic background, TDI, education and healthy diet, history of disease (hypertension, cardiovascular disease, diabetes, and stroke).

First quartile: 0.000 ≤ dietary biotin intake ≤ 32.133; second quartile: 32.133 < dietary biotin intake ≤ 40.540; third quartile: 40.540 < dietary biotin intake ≤ 50.765; fourth quartile: 50.765 < dietary biotin intake ≤ 101.459.

**Table S7.** Associations Between dietary biotin intake and anxiety and depression in participants with a typical diet.(n = 132,227).^a^

|  | **Dietary biotin intake** | | | | | | | | |
| --- | --- | --- | --- | --- | --- | --- | --- | --- | --- |
|  | **First quartile** | **Second quartile**  **HR (95%CI), *P*-value** | | | **Third quartile**  **HR (95%CI),*P*-value** | | | **Forth quartile**  **HR (95%CI),*P*-value** | |
| Anxiety or Depression disorders |  |  | | |  | | |  | |
| Number of cases/person- years | 3113 / 442609 | 2599 / 448686 | | | 2515 / 449740 | | | 2587 / 447521 | |
| Multivariate model^b^ | 1 (reference) | 0.87(0.82,0.91) | **<0.001** | 0.86(0.81,0.91) | | **<0.001** | 0.87(0.82,0.93) | | **<0.001** |
| Anxiety |  |  | | |  | | |  | |
| Number of cases/person- years | 1797 / 451924 | 1543 / 455950 | | | 1446 / 457076 | | | 1435 / 455680 | |
| Multivariate model^b^ | 1 (reference) | 0.88(0.82,0.94) | **<0.001** | | 0.84(0.78,0.90) | **<0.001** | | 0.83(0.77,0.91) | **<0.001** |
| Depression |  |  | | |  | | |  | |
| Number of cases/person- years | 2063 / 448560 | 1638 / 454109 | | | 1626 / 454610 | | | 1704 / 452399 | |
| Multivariate model^b^ | 1 (reference) | 0.85(0.79,0.91) | **<0.001** | | 0.87(0.81,0.93) | **<0.001** | | 0.89(0.83,0.97) | **0.004** |
| Comorbidity |  |  | | |  | | |  | |
| Number of cases/person- years | 747 / 457875 | 582 / 461373 | | | 557 / 461946 | | | 552 / 460558 | |
| Multivariate model^b^ | 1 (reference) | 0.84(0.75,0.94) | **0.003** | | 0.84(0.75,0.95) | **0.004** | | 0.84(0.73,0.95) | **0.007** |

a Results were presented HR and 95% CI

b Model was was adjusted age, sex, smoking status, drinking status, BMI, energy intake, ethnic background, TDI, education and healthy diet, history of

disease (hypertension, cardiovascular disease, diabetes, and stroke).

First quartile: 0.000 ≤ dietary biotin intake ≤ 31.895; second quartile: 31.895 < dietary biotin intake ≤ 40.265; third quartile: 40.265 < dietary biotin intake ≤

50.587; fourth quartile: 50.587 < dietary biotin intake ≤ 102.399.

Bold represents P-value <0.05.

**Table S8.** Associations Between dietary biotin intake and anxiety and depression after excluding participants who took substances for anxiety or depression at baseline(n = 118,535).^a^

|  | **Dietary biotin intake** | | | | | | | |
| --- | --- | --- | --- | --- | --- | --- | --- | --- |
|  | **First quartile** | **Second quartile**  **HR (95%CI), *P*-value** | | **Third quartile**  **HR (95%CI),*P*-value** | | | **Forth quartile**  **HR (95%CI),*P*-value** | |
| Anxiety or Depression disorders |  |  | |  | | |  | |
| Number of cases/person- years | 2372 / 396733 | 1808 / 403733 | | 1717 / 404187 | | | 1800 / 402901 | |
| Multivariate model^b^ | 1 (reference) | 0.79(0.74,0.84) | **<0.001** | 0.77(0.72,0.83) | **<0.001** | 0.80(0.74,0.86) | | **<0.001** |
| Anxiety |  |  | |  | | |  | |
| Number of cases/person- years | 1430 / 403150 | 1098 / 408363 | | 1026 / 408844 | | | 1047 / 407956 | |
| Multivariate model^b^ | 1 (reference) | 0.79(0.73,0.86) | **<0.001** | 0.75(0.69,0.82) | **<0.001** | | 0.77(0.70,0.84) | **<0.001** |
| Depression |  |  | |  | | |  | |
| Number of cases/person- years | 1501 / 401586 | 1083 / 407526 | | 1065 / 407571 | | | 1126 / 406516 | |
| Multivariate model^b^ | 1 (reference) | 0.77(0.71,0.84) | **<0.001** | 0.79(0.73,0.86) | **<0.001** | | 0.81(0.74,0.89) | **0.004** |
| Comorbidity |  |  | |  | | |  | |
| Number of cases/person- years | 559 / 408003 | 373 / 412156 | | 374 / 412228 | | | 373 / 411571 | |
| Multivariate model^b^ | 1 (reference) | 0.72(0.63,0.82) | **0.003** | 0.76(0.66,0.87) | **0.004** | | 0.74(0.63,0.87) | **0.007** |

a Results were presented HR and 95% CI

b Model was was adjusted age, sex, smoking status, drinking status, BMI, energy intake, ethnic background, TDI, education and healthy diet, history of

disease (hypertension, cardiovascular disease, diabetes, and stroke).

First quartile: 0.000 ≤ dietary biotin intake ≤ 31.961; second quartile: 31.961 < dietary biotin intake ≤ 40.428; third quartile: 40.428 < dietary biotin intake ≤

50.705; fourth quartile: 50.705< dietary biotin intake ≤ 101.460.

Bold represents P-value <0.05.


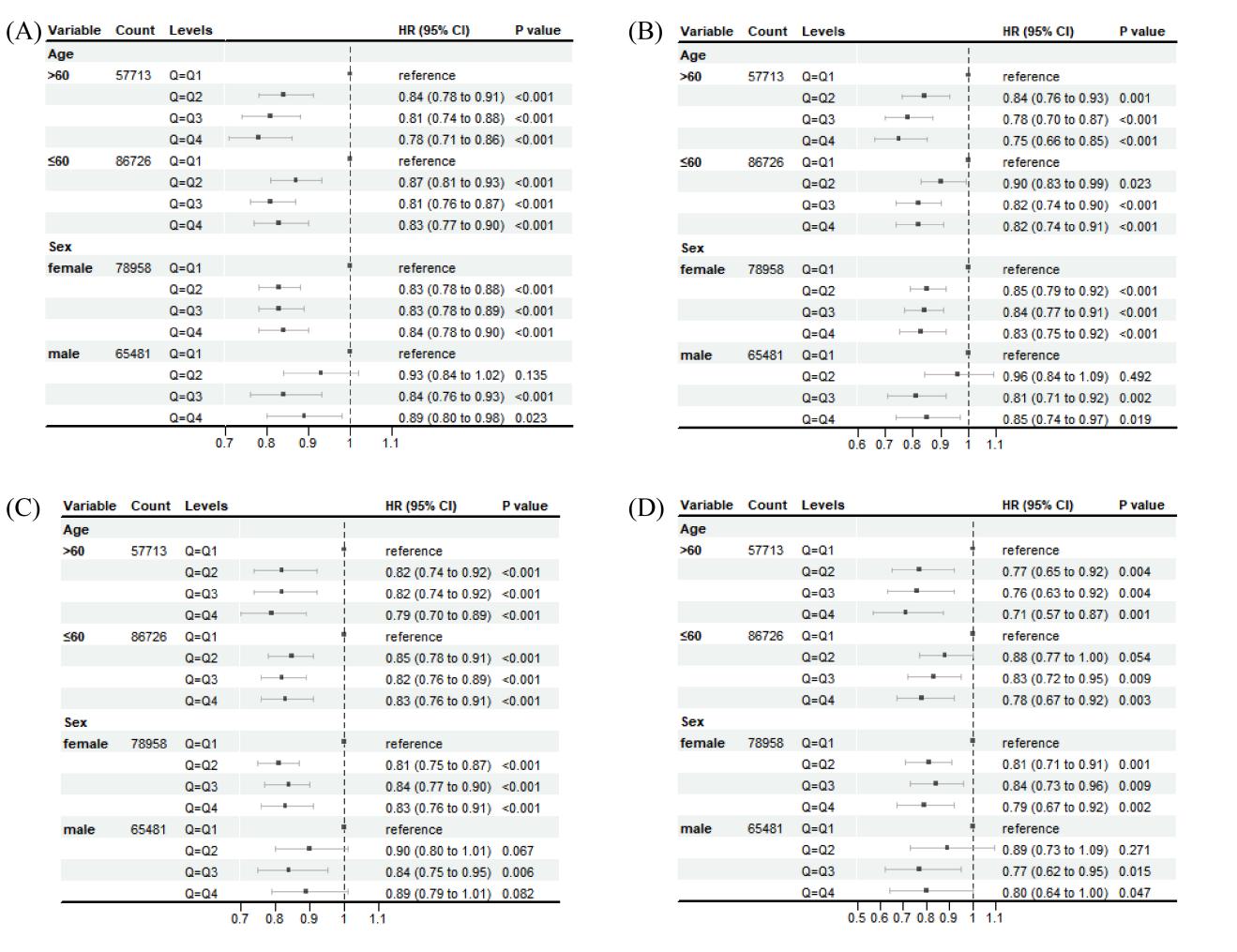


**Fig.S1** Associations between dietary biotin intake and (A)Anxiety or Depression, (B)Anxiety, (C)Depression, (D)Comorbidity across subgroups. Hazard ratio for each quantile increases in dietary biotin intake. Adjusted for age, sex, smoking status, drinking status, BMI, energy intake, ethnic background, TDI, education and healthy diet, history of disease (hypertension, cardiovascular disease, diabetes, and stroke).


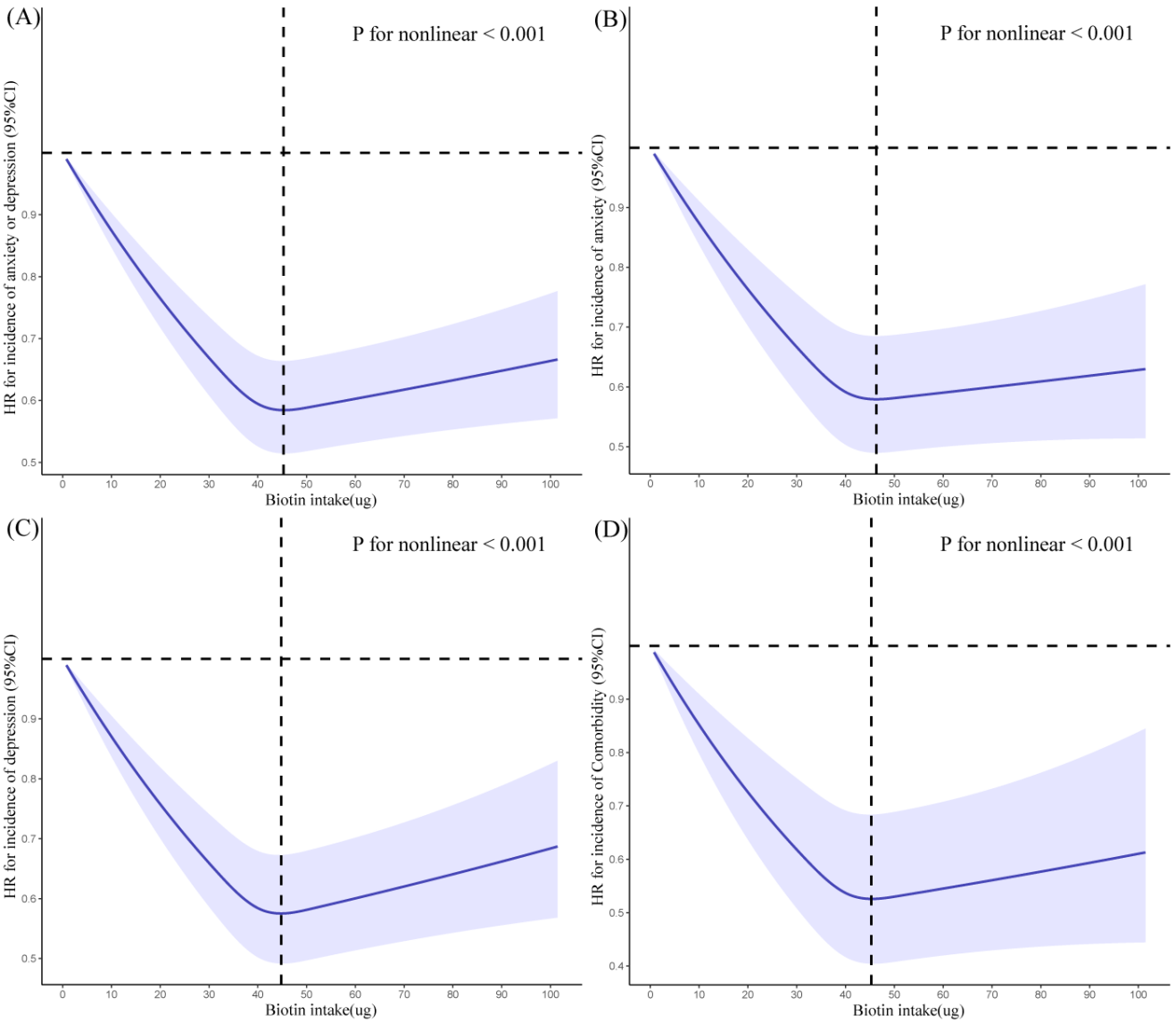


**Fig.S2** Restricted cubic spline for testing the hypothesis of nonlinear correlation between(A)Anxiety or Depression, (B)Anxiety, (C)Depression, (D)Comorbidity and dietary biotin intake after excluding participants who developed any dementia within the first 2 years. Spline curves represent hazard ratios (HRs) adjusted for age, sex, smoking status, drinking status, BMI, energy intake, ethnic background, TDI, education and healthy diet, history of disease(hypertension, cardiovascular disease, diabetes, and stroke).The solid lines are fitted based on Cox-proportional hazard models. The shaded areas show 95% confidential intervals (CIs). The black vertical line indicates the position where the curve inflection point occurs.


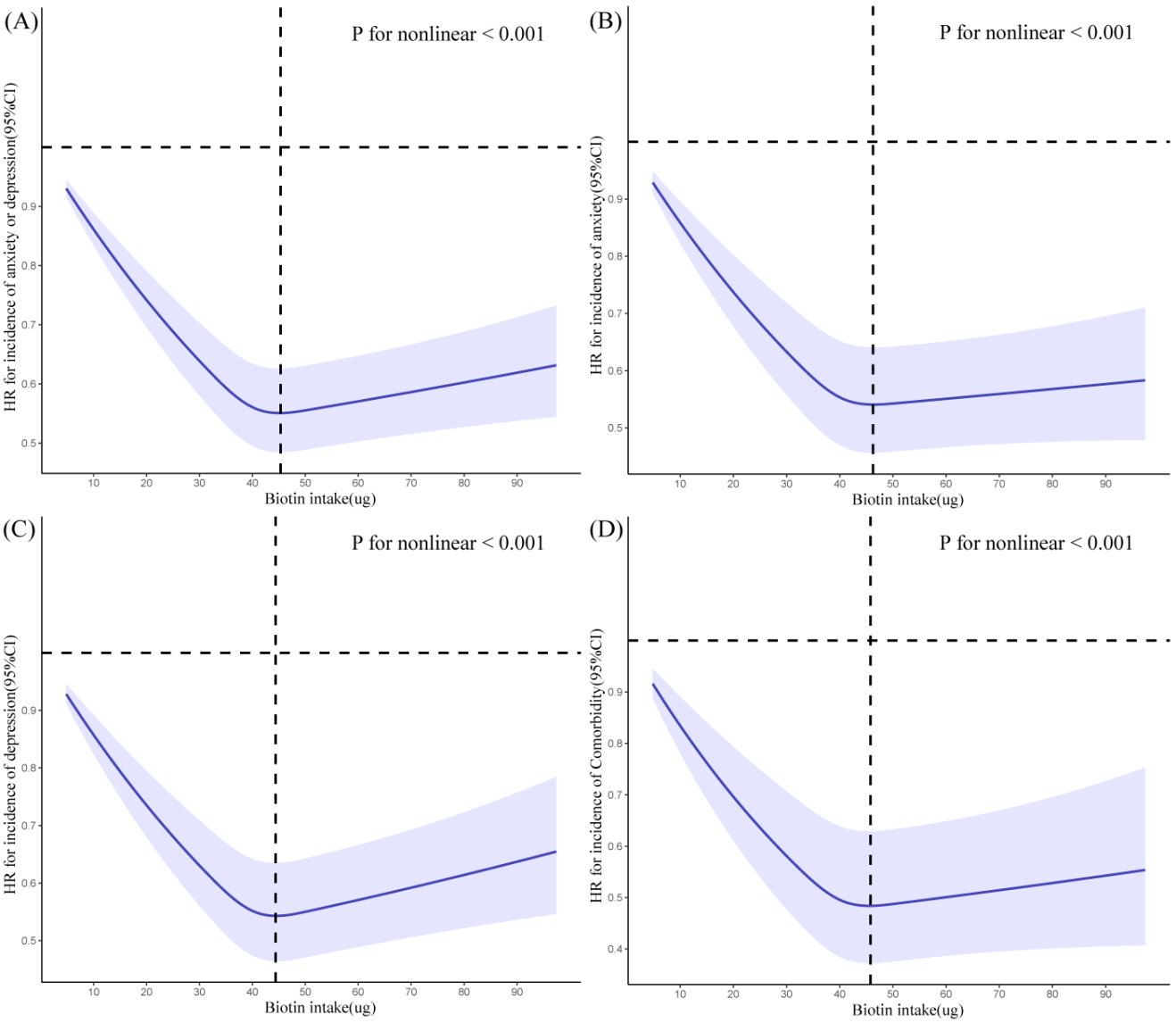


**Fig.S3** Restricted cubic spline for testing the hypothesis of nonlinear correlation between(A)Anxiety or Depression, (B)Anxiety, (C)Depression, (D)Comorbidity and dietary biotin intake after excluding unreliable data on energy intake. Spline curves represent hazard ratios (HRs) adjusted for age, sex, smoking status, drinking status, BMI, energy intake, ethnic background, TDI, education and healthy diet, history of disease(hypertension, cardiovascular disease, diabetes, and stroke).The solid lines are fitted based on Cox-proportional hazard models. The shaded areas show 95% confidential intervals (CIs). The black vertical line indicates the position where the curve inflection point occurs.


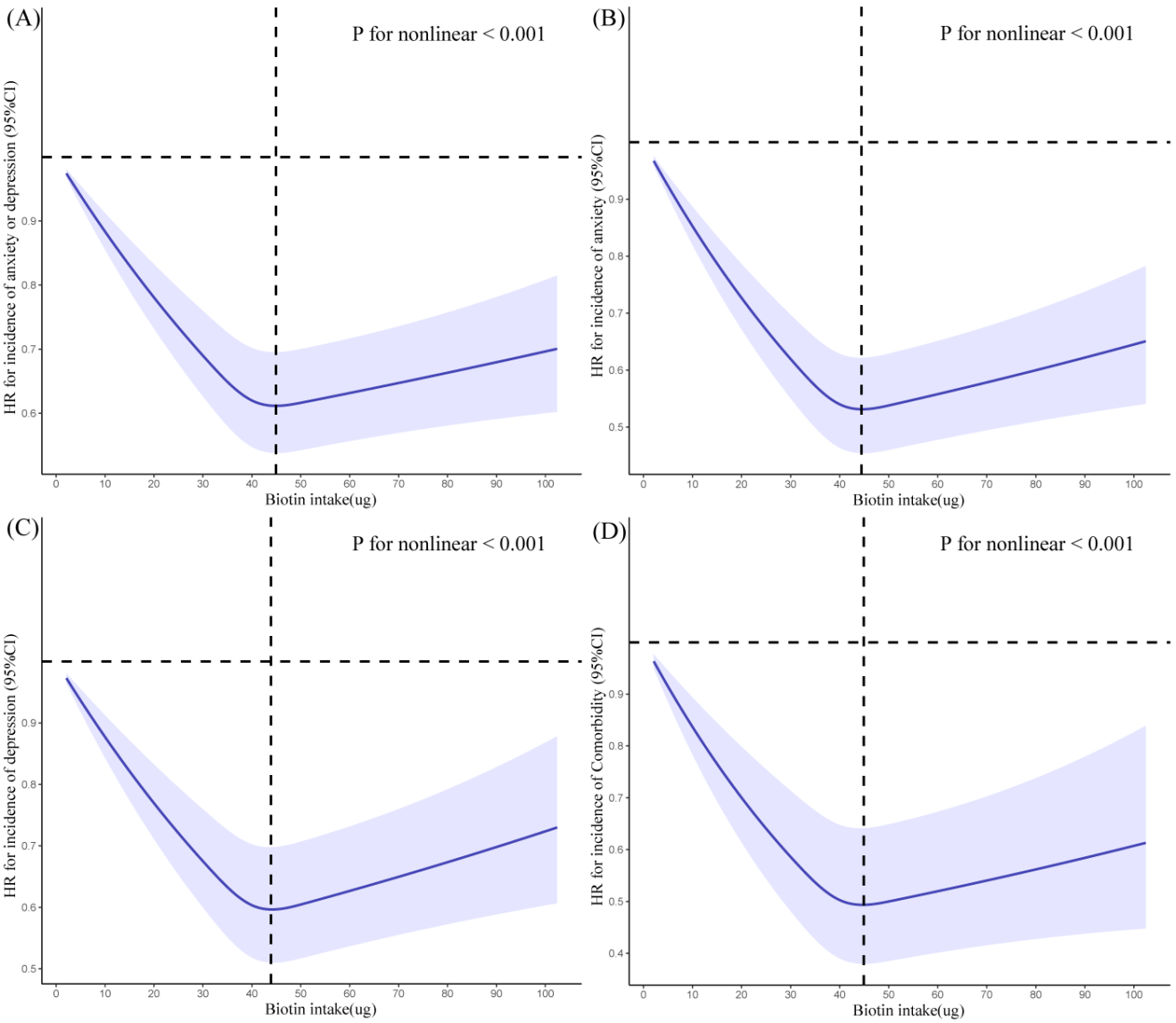


**Fig.S4** Restricted cubic spline for testing the hypothesis of nonlinear correlation between(A)Anxiety or Depression, (B)Anxiety, (C)Depression, (D)Comorbidity and dietary biotin intake in participants with a typical diet. Spline curves represent hazard ratios (HRs) adjusted for age, sex, smoking status, drinking status, BMI, energy intake, ethnic background, TDI, education and healthy diet, history of disease(hypertension, cardiovascular disease, diabetes, and stroke).The solid lines are fitted based on Cox-proportional hazard models. The shaded areas show 95% confidential intervals (CIs). The black vertical line indicates the position where the curve inflection point occurs.

**
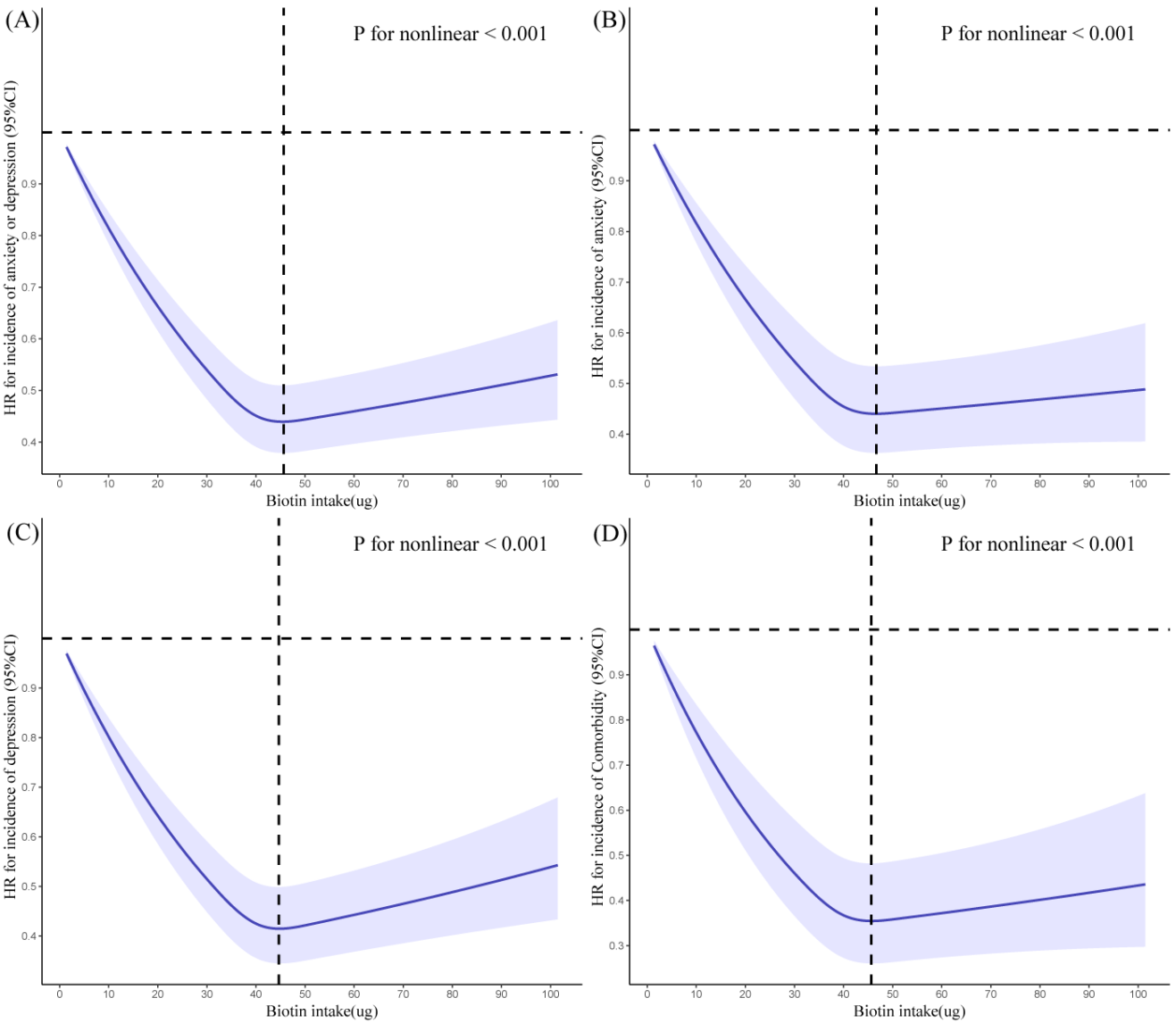
**

**Fig.S5** Restricted cubic spline for testing the hypothesis of nonlinear correlation between(A)Anxiety or Depression, (B)Anxiety, (C)Depression, (D)Comorbidity and dietary biotin intake after excluding participants who took substances for anxiety or depression at baseline. Spline curves represent hazard ratios (HRs) adjusted for age, sex, smoking status, drinking status, BMI, energy intake, ethnic background, TDI, education and healthy diet, history of disease(hypertension, cardiovascular disease, diabetes, and stroke).The solid lines are fitted based on Cox-proportional hazard models. The shaded areas show 95% confidential intervals (CIs). The black vertical line indicates the position where the curve inflection point occurs.


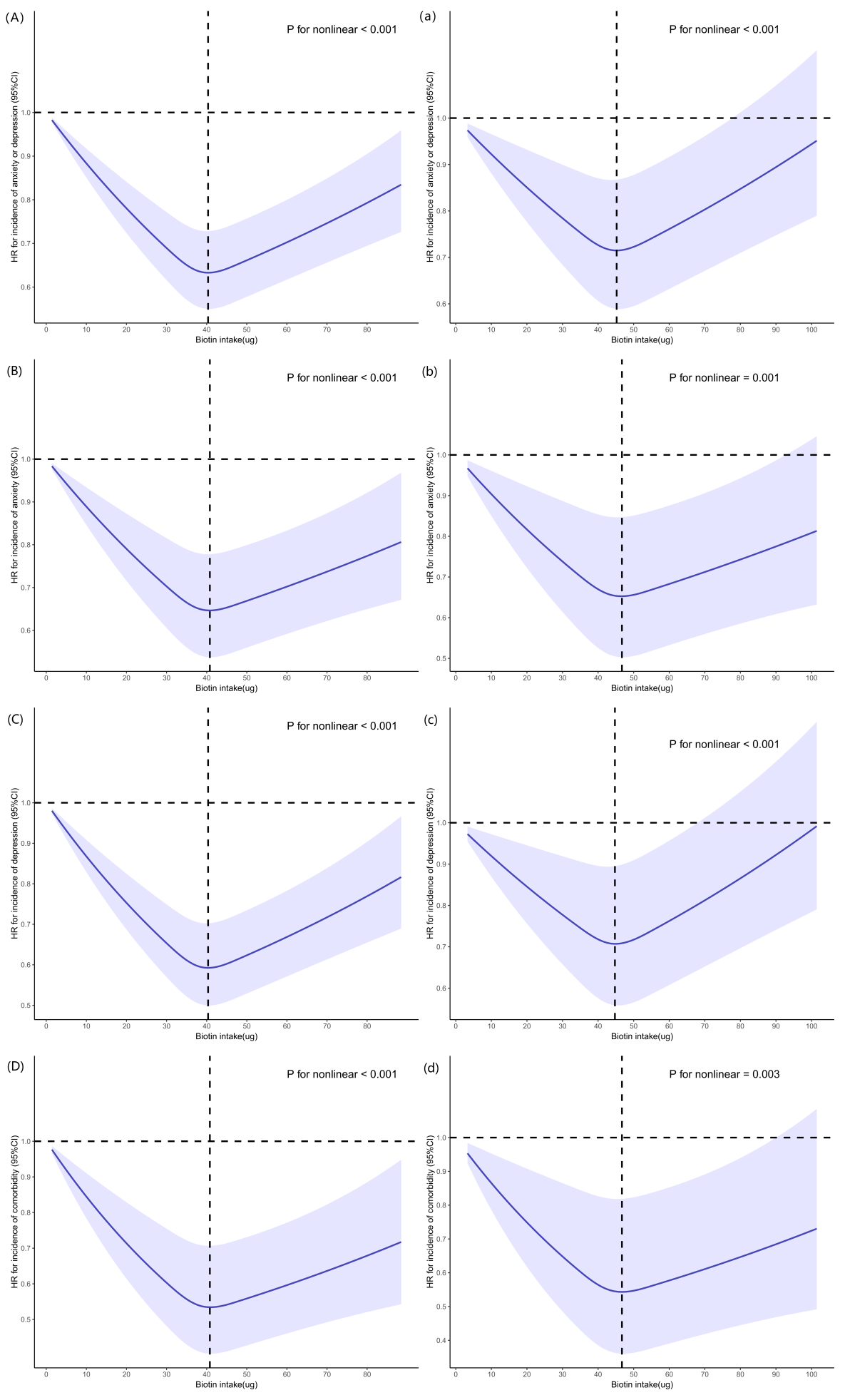


**Fig.S6** Restricted cubic spline for testing the hypothesis of nonlinear correlation between Anxiety or Depression in female(A)/male(a), Anxiety in female(B)/male(b), Depression in female(C)/male(c), Comorbidity in female(D)/male(d) and dietary biotin intake after excluding participants who took substances for anxiety or depression at baseline. Spline curves represent hazard ratios (HRs) adjusted for age, sex, smoking status, drinking status, BMI, energy intake, ethnic background, TDI, education and healthy diet, history of disease(hypertension, cardiovascular disease, diabetes, and stroke).The solid lines are fitted based on Cox-proportional hazard models. The shaded areas show 95% confidential intervals (CIs). The black vertical line indicates the position where the curve inflection point occurs.
